# Supplementary material for: Quantum error mitigation via quantum-noise-effect circuit groups
Source: Sci Rep. 2024 Mar 13;14:6077. doi: 10.1038/s41598-024-52485-7 (PMC11636875; doi:10.1038/s41598-024-52485-7)
Supplement: Supplementary file 1 — Supplementary Information. [file 41598_2024_52485_MOESM1_ESM.pdf]

Supplementary Information: Quantum Error Mitigation via Quantum-Noise-Effect Circuit Groups

Yusuke Hama*[1] and Hirofumi Nishi[2,1]

[1]*Quemix Inc., 2-11-2 Nihombashi, Chuo-ku, Tokyo 103-0027, Japan**

[2]*Laboratory for Materials and Structures, Institute of Innovative Research, Tokyo Institute of Technology, Yokohama 226-8503, Japan*

This supplementary information presents our extended QEM formalisms, the optimized variational parameters of QAOA, and the quantum circuit for the operation O^{QAA} given in Eq. (14) in the main text. Furthermore, it presents measurement data of T_1 and T_2 times and device properties of IBM Q Experience processors [1].

I. EXTENDED QEM FORMALISMS

In this section, we discuss extensions of our QEM formalism to various types of quantum noise effects; generalized amplitude damping (GAD), phase damping (PD), and their composite channels. Further, we discuss extensions to cases of higher-order quantum noise effects.

A. QEM for Other Quantum Noises

By taking exactly the same approach for deriving the QEM scheme for the AD effects we can straightforwardly construct our QEM schemes for other quantum noises including GAD, PD, their composite channels, and stochastic Pauli noises. First let us show the case of GAD effects. The quantum master equation for describing the GAD process is given by

$$\frac{\partial \rho(t)}{\partial t} = \gamma \mathcal{L}_{\mathrm{GAD}}[\rho(t)] = \gamma(\bar{n}+1) \sum_{j=0}^{N_q-1} \left[\tilde{\sigma}_j^- \rho(t) \tilde{\sigma}_j^+ - \frac{1}{2}\{\tilde{\sigma}_j^+ \tilde{\sigma}_j^-, \rho(t)\} \right] + \gamma \bar{n} \sum_{j=0}^{N_q-1} \left[\tilde{\sigma}_j^+ \rho(t) \tilde{\sigma}_j^- - \frac{1}{2}\{\tilde{\sigma}_j^- \tilde{\sigma}_j^+, \rho(t)\} \right], \quad (1)$$

where $\mathcal{L}_{\mathrm{GAD}}$ is the Lindblad superoperator of the GAD process and $\bar{n} = (e^{\beta\epsilon} - 1)^{-1}$ is the Bose-Einstein distribution function for the energy ϵ. The quantity β is the inverse temperature. Note that by taking the zero-temperature limit $(\beta \to \infty)$ in Eq. (1), we obtain the quantum master equation (1) in the main text. As we did in the case of the AD effect, let us estimate the GAD effect in the first order in τ and write it by $\Delta_1^{\mathrm{GAD}} \rho_{d\cdots 1}$ like $\Delta_1^{\mathrm{AD}} \rho_{d\cdots 1}$ in Eq. (2) in the main text. From the quantum master equation (1), the GAD effect $\Delta_1^{\mathrm{GAD}} \rho_{d\cdots 1}$ is evaluated as

$$\Delta_1^{\mathrm{GAD}} \rho_{d\cdots 1} = \sum_{k=1}^{d} \Delta_{1,k}^{\mathrm{GAD}} \rho_{d\cdots 1},$$
$$\Delta_{1,k}^{\mathrm{GAD}} \rho_{d\cdots 1} = \left(\prod_{l=k+1}^{d} U_l \right) \cdot \tilde{\rho}_{k\cdots 1}^{\mathrm{GAD}} \cdot \left(\prod_{l=k+1}^{d} U_l \right)^\dagger, \quad (2)$$

where

$$\begin{aligned} \tilde{\rho}_{k\cdots 1}^{\mathrm{GAD}} &= \mathcal{L}_{\mathrm{GAD}}[\rho_{k\cdots 1}] \\ &= \sum_{j=0}^{N_q-1} (\bar{n}+1) \left[\tilde{\sigma}_j^- \rho_{k\cdots 1} \tilde{\sigma}_j^+ - \frac{1}{2}\{P_j^1, \rho_{k\cdots 1}\} \right] \\ &+ \bar{n} \left[\tilde{\sigma}_j^+ \rho_{k\cdots 1} \tilde{\sigma}_j^- - \frac{1}{2}\{P_j^0, \rho_{k\cdots 1}\} \right] \\ &= (\bar{n}+1) \tilde{\rho}_{k\cdots 1}^{\mathrm{GAD,EM}} + \bar{n} \tilde{\rho}_{k\cdots 1}^{\mathrm{GAD,AB}}. \end{aligned} \quad (3)$$

* yhama@quemix.com

Physically, the quantity $\tilde{\rho}^{\text{GAD,EM}}_{k\cdots 1}$ in the above equation describes emission process of the register bits whereas $\tilde{\rho}^{\text{GAD,AB}}_{k\cdots 1}$ represents absorption process. Like Eq. (9) in the main text, we rewrite these two quantities in terms of the identity operator, Z gate, and the non-unitary operators $\{P^0, P^1, \tilde{\sigma}^{\pm}\}$. As a result, we have

$$\begin{aligned}\tilde{\rho}^{\text{GAD,EM}}_{k\cdots 1} &= \frac{-\rho_{k\cdots 1} + Z_j\rho_{k\cdots 1}Z_j + \tilde{\sigma}^-_j\rho_{k\cdots 1}\tilde{\sigma}^+_j - P^1_j\rho_{k\cdots 1}P^1_j}{4},\\ \tilde{\rho}^{\text{GAD,AB}}_{k\cdots 1} &= \frac{-\rho_{k\cdots 1} + Z_j\rho_{k\cdots 1}Z_j + \tilde{\sigma}^+_j\rho_{k\cdots 1}\tilde{\sigma}^-_j - P^0_j\rho_{k\cdots 1}P^0_j}{4}.\end{aligned} \tag{4}$$

Note that in the zero-temperature limit ($\beta \to \infty$) we have $\bar{n} \to 0$ and $\tilde{\rho}^{\text{GAD,EM}}_{k\cdots 1} \to \tilde{\rho}^{\text{AD}}_{k\cdots 1}$. By using Eq. (6) in the main text with setting $\vartheta = \pi$, the operation of $\tilde{\sigma}^+_j$ can be generated by the AD-effect circuit B with post-selecting the measurement outcome of the ancilla bit to be $|1\rangle$ while the operation of P^0_j is created by the AD-effect circuit A with post-selecting the output state $|0\rangle_{Q_\text{a}}$. Thus, as in the case of the AD effect, we can perform QEM for GAD by four types of quantum circuits mentioned previously, the original quantum circuit given by U^{QC}, the quantum circuits with the additional Z-gate operations, and the AD-effect circuits A and B. Therefore, the quantum-noise-effect circuit group for GAD effect is equivalent to that for AD effect and the maximum number of the quantum circuits which we need to perform QEM for GAD effect is $3dN_q + 1$. The QEM formula for the GAD effect is given by

$$\langle\hat{O}\rangle^{\text{QEM}}_{\rho_{d\cdots 1}} = \langle\hat{O}\rangle_{\rho_{d\cdots 1}} + \tau\Big(\langle\hat{O}\rangle_{\delta^{\text{GAD}}_1(\rho_{d\cdots 1})} - \langle\hat{O}\rangle_{\Delta^{\text{GAD}}_1\rho_{d\cdots 1}}\Big), \tag{5}$$

where the symbol δ^{GAD}_1 describes the GAD effect on a real device in the first order in τ and

$$\langle\hat{O}\rangle_{\Delta^{\text{GAD}}_1\rho_{d\cdots 1}} = (\bar{n}+1)\langle\hat{O}\rangle_{\Delta^{\text{GAD,EM}}_1\rho_{d\cdots 1}} + \bar{n}\langle\hat{O}\rangle_{\Delta^{\text{GAD,AB}}_1\rho_{d\cdots 1}}. \tag{6}$$

Our QEM scheme can also be extended to the case when both GAD and phase damping (PD) occur. The quantum master equation which describes such a process is given by

$$\begin{aligned}\frac{\partial\rho(t)}{\partial t} &= \gamma\mathcal{L}_{\text{GAD}}[\rho(t)] + \gamma_{\text{PD}}\mathcal{L}_{\text{PD}}[\rho(t)]\\ &= \gamma(\bar{n}+1)\sum_{j=0}^{N_q-1}\left[\tilde{\sigma}^-_j\rho(t)\tilde{\sigma}^+_j - \frac{1}{2}\{\tilde{\sigma}^+_j\tilde{\sigma}^-_j, \rho(t)\}\right]\\ &\quad + \gamma\bar{n}\sum_{j=0}^{N_q-1}\left[\tilde{\sigma}^+_j\rho(t)\tilde{\sigma}^-_j - \frac{1}{2}\{\tilde{\sigma}^-_j\tilde{\sigma}^+_j, \rho(t)\}\right]\\ &\quad + \gamma_{\text{PD}}\sum_{j=0}^{N_q-1}\left[Z_j\rho(t)Z_j - \rho(t)\right],\end{aligned} \tag{7}$$

where $\mathcal{L}_{\text{PD}}$ is the Lindblad superoperator of PD process and γ_{PD} is the strength of PD. We introduce the dimensionless quantity defined by $\tau_{\text{PD}} = \gamma_{\text{PD}} \cdot \Delta t$ and assume to be in the same order as τ. Like Eq. (2) in the main text, we represent the solution of quantum master equation (7) as the perturbative series of τ and τ_{PD} given by

$$\begin{aligned}\rho(T) &= \rho_{d\cdots 1} + \tau\Delta^{\text{GAD}}_1\rho_{d\cdots 1} + \tau_{\text{PD}}\Delta^{\text{PD}}_1\rho_{d\cdots 1}\\ &\quad + \mathcal{O}(\tau^2, \tau^2_{\text{PD}}, \tau\tau_{\text{PD}}),\end{aligned} \tag{8}$$

where $\Delta^{\text{PD}}_1\rho_{d\cdots 1}$ is

$$\begin{aligned}\Delta^{\text{PD}}_1\rho_{d\cdots 1} &= \sum_{k=1}^{d}\Delta^{\text{PD}}_{1,k}\rho_{d\cdots 1},\\ \Delta^{\text{PD}}_{1,k}\rho_{d\cdots 1} &= \left(\prod_{l=k+1}^{d}U_l\right)\tilde{\rho}^{\text{PD}}_{k\cdots 1}\left(\prod_{l=k+1}^{d}U_l\right)^{\dagger},\\ \tilde{\rho}^{\text{PD}}_{k\cdots 1} &= \mathcal{L}_{\text{PD}}[\rho_{k\cdots 1}] = \sum_{j=0}^{N_q-1}Z_j\rho_{k\cdots 1}Z_j - \rho_{k\cdots 1}.\end{aligned} \tag{9}$$

As we see in the above equation, to evaluate $\Delta_1^{\rm PD}\rho_{d\cdots 1}$ the only additional operation which we need is the Z-gate operation. Therefore, the quantum-noise-effect circuit group for the composite channel of the GAD and PD effects is equivalent to that of GAD (AD) effect. Let us denote the composition of the GAD and PD effects which occur on a real device by the symbol $\delta^{\rm GAD\cdot PD}$. In the first-order approximation with respect to τ and $\tau_{\rm PD}$ we can express $\delta^{\rm GAD\cdot PD}(\rho_{d\cdots 1})$ as $\delta^{\rm GAD\cdot PD}(\rho_{d\cdots 1}) = \tau\delta_1^{\rm GAD}(\rho_{d\cdots 1}) + \tau_{\rm PD}\delta_1^{\rm PD}(\rho_{d\cdots 1})$. Our QEM formula is given by

$$\begin{aligned}\langle\hat{O}\rangle_{\rho_{d\cdots 1}}^{\rm QEM} &\equiv \langle\hat{O}\rangle_{\rho_{d\cdots 1}^{\rm real}} - \tau\langle\hat{O}\rangle_{(\Delta_1^{\rm GAD}\rho_{d\cdots 1})^{\rm real}} - \tau_{\rm PD}\langle\hat{O}\rangle_{(\Delta_1^{\rm PD}\rho_{d\cdots 1})^{\rm real}} \\ &= \langle\hat{O}\rangle_{\rho_{d\cdots 1}} + \tau\left(\langle\hat{O}\rangle_{\delta_1^{\rm GAD}(\rho_{d\cdots 1})} - \langle\hat{O}\rangle_{\Delta_1^{\rm GAD}\rho_{d\cdots 1}}\right) + \tau_{\rm PD}\left(\langle\hat{O}\rangle_{\delta_1^{\rm PD}(\rho_{d\cdots 1})} - \langle\hat{O}\rangle_{\Delta_1^{\rm PD}\rho_{d\cdots 1}}\right) + \mathcal{O}(\tau^2, \tau_{\rm PD}^2, \tau\tau_{\rm PD}). \end{aligned} \tag{10}$$

We end this section by noting that our QEM scheme can be extended to the mitigation of stochastic Pauli noise effects (bit flip, phase flip, bit-phase flip, depolarizing channel): Here we do not show detailed analysis for them. Like the PD effect, the stochastic Pauli noise effects are represented by the identity operator and X, Y, and Z gates. For these cases, to conduct QEM we just need to prepare quantum circuits composed with additional X, Y, and Z gate operations like $\sum_{j=0}^{N_q-1} Z_j\rho_{k\cdots 1}Z_j$ in Eq. (9) in the main text and the ancilla bits are not needed.

B. QEM for Higher-Order AD Effects

When a quantum state at a time t_0, which we denote by $\rho(t_0)$, is subject to the AD effect during the time interval Δt, according to the quantum master equation (1) in the main text the quantum state at the time $t = t_0 + \Delta t$ is given by $\rho(t_0 + \Delta t) = \rho(t_0) + \mathcal{L}_{\rm AD}[\rho(t_0)]\tau + \mathcal{L}_{\rm AD}^2[\rho(t_0)]\frac{\tau^2}{2!} + \mathcal{O}(\tau^3)$. Here $\mathcal{L}_{\rm AD}^2$ describes the twice operation of $\mathcal{L}_{\rm AD}$ and it is equivalent to the second-order time derivate obeying the quantum master equation (1) in the main text. In other words, we have solved the quantum master equation (1) in the main text up to the second order in Δt. Let us analyze the quantum state generated by the unitary operation $U^{\rm QC} = \prod_{k=1}^d U_k = U_d \cdot U_{d-1}\cdots U_2\cdot U_1$ under the influence of the AD effect and write it by $\rho(T)$. As similar to the second line of the right-hand side of Eq. (2) in the main text, up to $\mathcal{O}(\tau^2)$ the density matrix $\rho(T)$ is calculated as

$$\rho(T) = \rho_{d\cdots 1} + \tau\Delta_1^{\rm AD}\rho_{d\cdots 1} + \frac{\tau^2}{2!}\Delta_2^{\rm AD}\rho_{d\cdots 1} + \mathcal{O}(\tau^3), \tag{11}$$

where $\Delta_2^{\rm AD}\rho_{d\cdots 1}$ denotes the theoretically-evaluated second-order AD effect associated with the unitary operation $U^{\rm QC}$ and is given by

$$\begin{aligned}\Delta_2\rho_{d\cdots 1} =& \sum_{k=1}^{d}\sum_{j_1,j_2=0}^{N_q-1}\left(\prod_{l=k+1}^{d}U_l\right)\mathcal{L}_{j_1}^{\rm AD}\left[\mathcal{L}_{j_2}^{\rm AD}[\rho_{k\cdots 1}]\right]\left(\prod_{l=k+1}^{d}U_l\right)^\dagger \\ &+ 2\sum_{k_1=2}^{d-1}\sum_{k_2=1}^{k_1-1}\sum_{j_1,j_2=0}^{N_q-1}\left(\prod_{l_1=k_1+1}^{d}U_{l_1}\right)\mathcal{L}_{j_1}^{\rm AD}\left[\left(\prod_{l_2=k_2+1}^{k_1}U_{l_2}\right)\mathcal{L}_{j_2}^{\rm AD}[\rho_{k_2\cdots 1}]\left(\prod_{l_2=k_2+1}^{k_1}U_{l_2}\right)^\dagger\right]\left(\prod_{l_1=k_1+1}^{d}U_{l_1}\right)^\dagger. \end{aligned} \tag{12}$$

The term in the first line of the right-hand side corresponds to the second-order derivative. By using Eq. (9) in the main text, $\Delta_2\rho_{d\cdots 1}$ in Eq. (12) is represented by the operators $\{\mathbf{1}_{2\times 2}, Z, \tilde{\sigma}^-, P^1\}$ as

$$\begin{aligned}\Delta_2\rho_{d\cdots 1} =& \sum_{k=1}^{d}\sum_{j_1,j_2}^{N_q-1}\sum_{p_1,p_2}c_{p_1}c_{p_2}\left(\prod_{l=k+1}^{d}U_l\right)s_{Q_{j_1},p_1}s_{Q_{j_2},p_2}\rho_{k\cdots 1}s_{Q_{j_2},p_2}^\dagger s_{Q_{j_1},p_1}^\dagger\left(\prod_{l=k+1}^{d}U_l\right)^\dagger \\ &+ 2\sum_{k_1=2}^{d}\sum_{k_2=1}^{k_1-1}\sum_{j_1,j_2}^{N_q-1}\sum_{p_1,p_2}c_{p_1}c_{p_2}\left(\prod_{l_1=k_1+1}^{d}U_{l_1}\right)s_{Q_{j_1},p_1}\left(\prod_{l_2=k_2+1}^{k_1}U_{l_2}\right)s_{Q_{j_2},p_2}\rho_{k_2\cdots 1} \\ &\times s_{Q_{j_2},p_2}^\dagger\left(\prod_{l_2=k_2+1}^{k_1}U_{l_2}\right)^\dagger s_{Q_{j_1},p_1}^\dagger\left(\prod_{l_1=k_1+1}^{d}U_{l_1}\right)^\dagger, \end{aligned} \tag{13}$$

where the subscripts p_1 and p_2 are used for labeling the four operators $\{\mathbf{1}_{2\times 2}, Z, \tilde{\sigma}^-, P^1\}$, and correspondingly, $s_{Q_{j_a},p_a}$ $(a = 1, 2)$ is the operator acting on the qubit Q_{j_a} and $s_{Q_{j_a},p_a} \in \{\mathbf{1}_{2\times 2, j_a}, Z_{j_a}, \tilde{\sigma}_{j_a}^-, P_{j_a}^1\}$. For instance, $s_{Q_{j_a},Z}$ is the Z-gate operation acting on the qubit Q_{j_a}. The coefficient c_{p_a} $(a = 1, 2)$ is assigned when the operator $s_{Q_{j_a},p_a}$ is acted on the qubit

Q_{j_a}. The explicit form of the coefficients c_{p_a} are $\{c_{\mathbf{1}_{2\times2}}, c_Z, c_{\tilde{\sigma}^-}, c_{P^1}\} = \{-\frac{1}{4}, \frac{1}{4}, 1, -1\}$. By representing the second-order effect $\Delta_2\rho_{d\cdots1}$ in the way given in Eq. (13), we can programmably evaluate $\Delta_2\rho_{d\cdots1}$ by the unitary operators U_l $(l = 1, \ldots, d)$ which compose the quantum algorithm under consideration, the additional operations $s_{Q_{j_a},p_a} \in \{\mathbf{1}_{2\times2}, Z, \tilde{\sigma}^-, P^1\}$, and the coefficients $c_{p_a} \in \{-\frac{1}{4}, \frac{1}{4}, 1, -1\}$. In other words, we can create the AD-effect (quantum-noise-effect) circuit group for evaluating $\Delta_2\rho_{d\cdots1}$. Note that the quantum circuits for evaluating the term in the first line of Eq. (13) is equivalent to the AD-effect-circuit group for evaluating $\Delta_1\rho_{d\cdots1}$ for $j_1 = j_2$. By using Eq. (8) in the main text, we can derive the formula of QEM for the second-order AD effect and is given by

$$\begin{aligned}
\langle\hat{O}\rangle^{\rm QEM}_{\rho_{d\cdots1}} \equiv{}& \langle\hat{O}\rangle_{\rho^{\rm real}_{d\cdots1}} - \tau\langle\hat{O}\rangle_{(\Delta_1^{\rm AD}\rho_{d\cdots1})^{\rm real}} - \frac{\tau^2}{2!}\langle\hat{O}\rangle_{(\Delta_1^{\rm AD}\rho_{d\cdots1})^{\rm real}} + \tau^2\langle\hat{O}\rangle_{\Delta_1^{\rm AD}\left((\Delta_1^{\rm AD}\rho_{d\cdots1})\right)^{\rm real}} \\
={}& \langle\hat{O}\rangle_{\rho_{d\cdots1}} + \tau\Big(\langle\hat{O}\rangle_{\delta_1^{\rm AD}(\rho_{d\cdots1})} - \langle\hat{O}\rangle_{\Delta_1^{\rm AD}\rho_{d\cdots1}}\Big) \\
&+ \frac{\tau^2}{2!}\Big(\langle\hat{O}\rangle_{\delta_2^{\rm AD}(\rho_{d\cdots1})} - \langle\hat{O}\rangle_{\Delta_2^{\rm AD}\rho_{d\cdots1}}\Big) - \tau^2\Big(\langle\hat{O}\rangle_{\delta_1^{\rm AD}\left((\Delta_1^{\rm AD}\rho_{d\cdots1})\right)} - \langle\hat{O}\rangle_{\Delta_1^{\rm AD}\left((\Delta_1^{\rm AD}\rho_{d\cdots1})\right)}\Big) + \mathcal{O}(\tau^3).
\end{aligned} \tag{14}$$

The first term in the second line of the right-hand side of the above equation is the ideal expectation value while the second term describes the QEM for the first-order AD effect; compare with Eq. (8) in the main text. On the other hand, the terms in the third line of the right-hand side describes the conduction of the QEM for the second-order AD effect. The first term describes the QEM for the intrinsic second-order AD effect while the second term describes the mitigation for the first-order AD effect $\delta_1^{\rm AD}\big((\Delta_1^{\rm AD}\rho_{d\cdots1})\big)$ and such an error has been neglected in the first-order perturbation theory. To calculate $\Delta_1^{\rm AD}\big((\Delta_1^{\rm AD}\rho_{d\cdots1})\big)$, first we need to rewrite the terms in $\Delta_1^{\rm AD}\rho_{d\cdots1}$ which are expressed by U_k and the single-time additional operations of $\tilde{\sigma}^-$ and P^1 as the terms given by the unitary operations $U_k \otimes \mathbf{1}_{Q_{\rm a}}$ and the gate operations composing the AD circuits A and B, respectively: see also Fig. 3 in the main text. Then we calculate $\Delta_1^{\rm AD}\big((\Delta_1^{\rm AD}\rho_{d\cdots1})\big)$ which is represented in the way that the operations $\{Z, \tilde{\sigma}^-, P^1\}$ are added for the series of unitary operations given by $U_k \otimes \mathbf{1}_{Q_{\rm a}}$, Z gate, and the quantum gates comprising the AD circuits A and B. Consequently, we have derived the QEM for the second-order AD effect and is represented by Eq. (14).

The above argument can be extended into the formulations of QEM schemes for high-order AD effects. By using Eq. (9) in the main text, we can evaluate the higher-order AD effects $\Delta_p^{\rm AD}\rho_{d\cdots1}$ $(p \geq 3)$ and represented them in programmable ways in terms of the operators $\{\mathbf{1}_{2\times2}, Z, \tilde{\sigma}^-, P^1\}$ like Eq. (13) and create p-th-order quantum-noise circuit groups. Then we can derive the QEM formula for the higher-order AD effects $\delta_p^{\rm AD}\rho_{d\cdots1}$. In order to do this, we need to evaluate not only $\Delta_p^{\rm AD}\rho_{d\cdots1}$ but also $\Delta_k^{\rm AD}\big(\Delta_{p-k}^{\rm AD}\rho_{d\cdots1}\big)$ such that $1 \leq k < p$, which is coming from the conduction of QEM for $p-k$-th order AD effects $\delta_k^{\rm AD}\rho_{d\cdots1}\big(\Delta_{p-k}^{\rm AD}\rho_{d\cdots1}\big)$. Furthermore, by applying the argument given above we can construct QEM formulas for higher-order effects of GAD, PD, their mixture, and the stochastic Pauli noises.

For evaluating the second-order effect $\Delta_2^{\rm AD}\rho_{d\cdots1}$, we perform the additional operators $\{Z, \tilde{\sigma}^-, P^1\}$ twice as shown in Eq. (13) and the number of additional quantum circuits to create a second-order AD-effect quantum circuit group is $\mathcal{O}(\{dN_q\}^2)$ and maximally we need two ancilla bits. Similarly, in the case of p-th order AD-effect circuit groups, we need to perform p additional operations of $\{Z, \tilde{\sigma}^\pm, P^0, P^1\}$, and correspondingly, we obtain the additional quantum circuits whose number is $\mathcal{O}(\{dN_q\}^p)$ and we need maximally p ancilla bits.

II. QUANTUM CIRCUIT FOR $O_{\rm QAA}$

In Fig. 1, we show the quantum circuit for $O_{\rm QAA}$ [33]

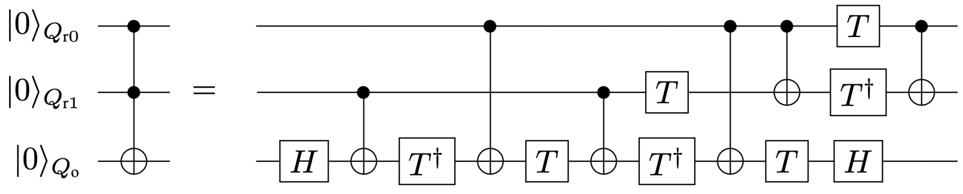

FIG. 1. Quantum circuit for $O^{\rm QAA}$ in Eq. (14).

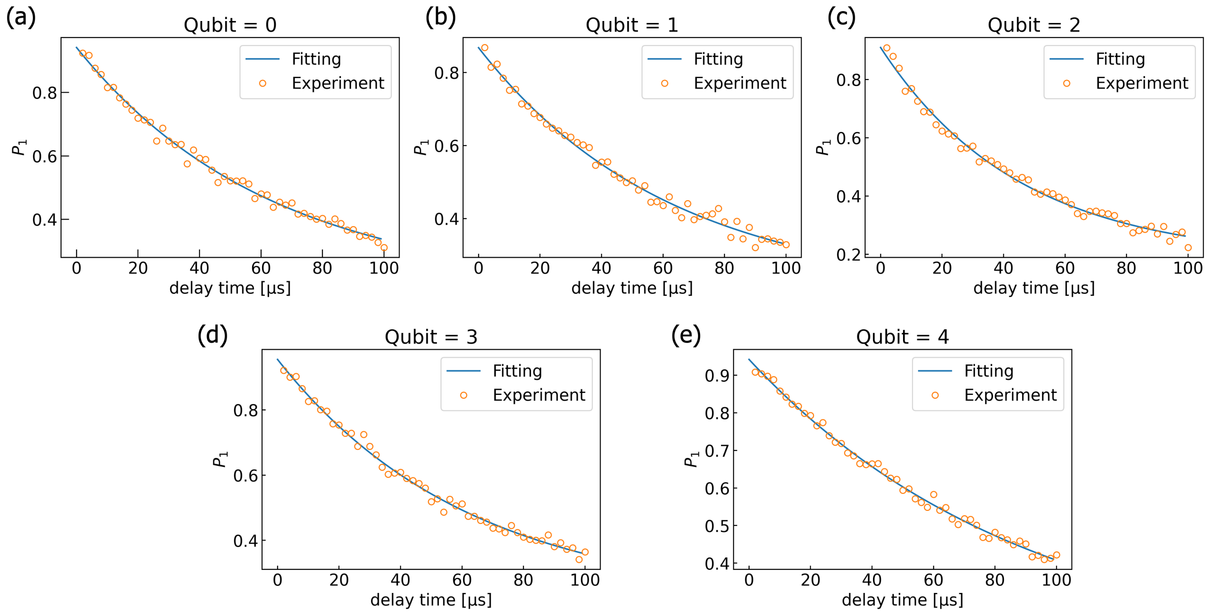

FIG. 2. Experimental data of the expectation values $\langle P^1\rangle(t_a^{\mathrm{tot},T_1})$ for the qubits Q_j $(j=0,\ldots,4)$ in ibmq_belem. The data was taken at 12:33, 09/11/2023.

III. OPTIMIZED VARIATIONAL PARAMETERS OF QAOA

We list the optimized numerical values of the variational parameters of QAOA which are given by

$$\begin{aligned}\vartheta_1^{\mathrm{QAOA}} &= 2.023075,\\ \vartheta_2^{\mathrm{QAOA}} &= 2.130055,\\ \varphi_1^{\mathrm{QAOA}} &= 1.011537,\\ \varphi_2^{\mathrm{QAOA}} &= 1.118518.\end{aligned} \tag{15}$$

IV. MEASUREMENT DATA OF T_1 AND T_2 TIMES

In this section, we explain how to extract the experimental values of T_1 and T_2 times with showing our data plots, Fig. 2 (T_1 data points) and Fig. 3 (T_2 data points). The experiment is done by ibmq_belem (five-qubit machine). We note that for both cases the relaxation processes given by the relaxation times t_a^{relax} were generated by using a function (command) called “delay" [1].

Let us explain from the measurements of the T_1 times. In Fig. 2, we plot the experimental data of the expectation values $\langle P^1\rangle(t_a^{\mathrm{tot},T_1})$ for the qubits Q_j $(j=0,\ldots,4)$. Here we have taken $t_a^{\mathrm{relax}} = 2\times a$ (μs) with $a=1,\ldots,50$ and $N_{\mathrm{QC}}=2^{10}$. We fit these data points by an exponential function $\langle P^1\rangle(t_a^{\mathrm{tot},T_1}) = \exp\left(-\frac{t_a^{\mathrm{tot},T_1}}{T_1}\right)$ and extract the values of T_1. In Table I we list these experimental values. In addition, we show the data which are open to the public in the parentheses [1]. As a result, our experimental data agrees well with them.

Next, let us discuss the measurement data of the T_2 times presented in Fig. 3. Here we plot the experimental data points of $\langle P^1\rangle(t_a^{\mathrm{tot},T_2})$ for the qubits Q_j and we have taken $t_a^{\mathrm{relax}} = \frac{16}{45}a$ (μs) with $a=1,\ldots,300$ and $N_{\mathrm{QC}}=2^{10}$. In this case, instead of a simple exponential decay curve, we fit the data points by a function

$$f_{T_2}(t_a^{\mathrm{tot},T_2}) = e^{-\frac{t_a^{\mathrm{tot},T_2}}{T_2}}\left[a_1\cos(2\pi f_1 t_a^{\mathrm{tot},T_2}+\phi_1)+a_2\cos(2\pi f_2 t_a^{\mathrm{tot},T_2}+\phi_2)\right]+b, \tag{16}$$

where $a_{1,2}, \phi_{1,2}$, and b are real numbers. The reasons we fit $\langle P^1\rangle(t_a^{\mathrm{tot},T_2})$ with $f_{T_2}(t_a^{\mathrm{tot},T_2})$ in Eq. (16) are the following. First, a detuning, which is a difference between the qubit frequency and a driving frequency (frequency of a pulse which generates a gate operation), is not actually zero, and instead of an exponential decay the plots of an expectation value of P^1 must be fitted by a damped oscillation curve [1]. Here we fit the the oscillation part by a cosine curve. When the detuning, however, is small it is

Qubit	T_1 (μs)	T_2 (μs)
Q_0	62.93 (109.91)	50.00 (178.67)
Q_1	62.31 (81.12)	89.38 (95.21)
Q_2	45.03 (79.47)	50.00 (60.13)
Q_3	61.95 (98.60)	78.29 (148.77)
Q_4	90.69 (93.07)	50.00 (146.12)

TABLE I. Experimental values of T_1 and T_2 times for ibmq_belem. The values in the parentheses are the data which are open to the public [1]. Our measurement data was taken at 12:33, 09/11/2023. The data open to the public were taken at 05:08, 09/11/2023.

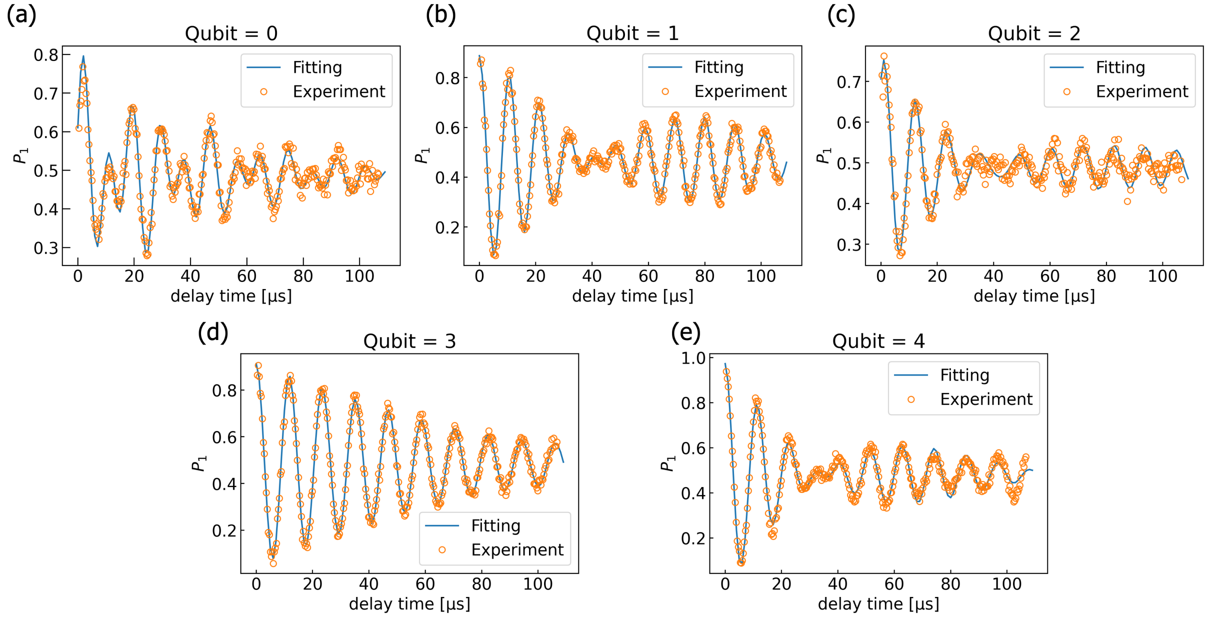

FIG. 3. Experimental data of the expectation values $\langle P^1\rangle(t_a^{\mathrm{tot},T_2})$ for the qubits Q_j ($j = 0, \dots, 4$) in ibmq_belem. The data was taken at 12:33, 09/11/2023.

not effective to fit the plots by the damped oscillation function since it is hard to distinguish whether the decreasing behaviors of the plots are coming from the exponential decay (decoherence) or from the oscillation. We avoid such an issue by enhancing the magnitude of the detuning explicitly. Thus, by writing the unitary operation for the measurement of the T_2 times by U^{meas,T_2}, the actual series of the gate operations which have been exploited for the T_2 measurements are not the two Hadamard gates but $U^{\mathrm{meas},T_2} = H \cdot \boldsymbol{I} \cdot R_z(\theta^{\mathrm{det}}) \cdot H$ with $\theta^{\mathrm{det}} = \frac{16a}{225}\pi$ ($a = 1, \dots, 300$). The rotation operation $R_z(\theta^{\mathrm{det}})$ plays the role of the enhancement of the detuning whereas $\boldsymbol{I}$ represents the relaxation part and is generated by the delay command as mentioned above. Second, we have examined that the fitting by a damped oscillation function with an oscillation part given by a single type of amplitude, frequency, and phase is not a good fitting, and instead, using the function $f_{T_2}(t_a^{\mathrm{tot},T_2})$ in Eq. (16), which is given by two types of amplitudes, frequencies, and phases, shows much better fitting. We consider the physical reasoning for this as follows. In [2], two different two-level systems having opposite charge parities induced by quasiparticle tunneling have been observed in a transmon qubit system, and in such a case there exist two different frequencies of the two-level systems. Since the ibmq_belem is a transmon qubit system [3, 4] we consider that the similar phenomenon has been observed in our experiment and we fitted the data points with the function $f_{T_2}(t_a^{\mathrm{tot},T_2})$ in Eq. (16). As a result, we obtain the values of $T_{2,j}$. In Table I, we display the experimental data of T_2 as well as the data open to the public [1] written in the parenthesis. The T_2 times for Q_1 and Q_2 show good agreement whereas those for the other qubits have moderate differences although the orders are the same.

V. QUANTUM DEVICE PROPERTIES

In this section, we present the data of the physical properties of ibmq_belem and ibm_perth (seven-qubit machine) which is open to the public [1]: In Fig. 4, we display illustrations of spatial configurations for qubits in these machines. First, we list the single-qubit properties of ibmq_belem, qubit frequencies and gate operation times in Table II. Here $t_{id}, t_{Rz}, t_{\sqrt{X}}$, and t_X

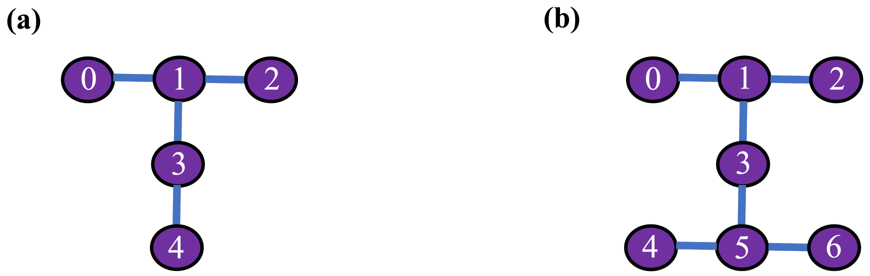

FIG. 4. Illustration of the spatial configuration for qubits for (a) ibmq_belem and (b) ibm_perth.

denote the gate times of identity gate, virtual Z gate, $\sqrt{X}$ gate, and X gate, respectively. In addition, we present the two-qubit gate properties, CNOT operation times denoted by t_{CX}. The symbol $[Q_i, Q_j]$ $(i, j = 0, 1, 2, 3, 4$ with $i \neq j)$ represent nearest-neighboring qubits Q_i and Q_j. The gate operation times in Table II and Table III are used for the implementation of our QEM for the quantum algorithm $U^{\rm QC}_{\rm imp,2}$: see the caption of Fig. 22 in the main text. Similarly, in Table IV-VII we list the physical

Qubit	ω_{01} (GHz)	t_{id} (ns)	t_{Rz} (ns)	$t_{\sqrt{X}}$ (ns)	t_X (ns)
Q_0	5.09	35.56	35.56	35.56	35.56
Q_1	5.25	35.56	35.56	35.56	35.56
Q_2	5.36	35.56	35.56	35.56	35.56
Q_3	5.17	35.56	35.56	35.56	35.56
Q_4	5.26	35.56	35.56	35.56	35.56

TABLE II. List of the single-qubit properties of ibmq_belem which are open to the public [1]. The above data was taken at 05:08, 09/11/2023.

Qubits	t_{CX} (ns)
$[Q_0, Q_1]$ $([Q_1, Q_0])$	810.67 (775.11)
$[Q_1, Q_2]$ $([Q_2, Q_1])$	419.56 (384.00)
$[Q_1, Q_3]$ $([Q_3, Q_1])$	440.89 (405.33)
$[Q_3, Q_4]$ $([Q_4, Q_3])$	526.22 (490.67)

TABLE III. List of the two-qubit properties of ibmq_belem which are open to the public [1]. The above data was taken at 05:08,09/11/2023.

properties of ibm_perth: Table IV and Table V present the data of the single-qubit properties while Table VI and Table VII show those of the two-qubit properties. We have used the data of the gate operation times taken in 09/26/2023 for the implementation of our QEM for the quantum algorithm $U^{\rm QC}_{\rm pre1}$ (Table V and Table VII) while we have used the ones taken in 09/21/2023 for $U^{\rm QC}_{\rm imp,2}$ (Table IV and Table VI): see also the captions of Fig. 20 and Fig. 21 in Sec. VB in the main text.

VI. REFERENCES

[1] Ibm quantum experience [online], Available at https://quantum-computing.ibm.com/ (2023).
[2] D. Ristè, C. Bultink, M. J. Tiggelman, R. N. Schouten, K. W. Lehnert, and L. DiCarlo, Nature communications **4**, 1913 (2013).
[3] E. A. C. Pérez, J. Bonitati, D. Lee, S. Quaglioni, and K. A. Wendt, Physical Review A **105**, 032403 (2022).
[4] D. Volya and P. Mishra, arXiv preprint arXiv:2302.13518 (2023).

Qubit	ω_{01} (GHz)	t_{id} (ns)	t_{Rz} (ns)	$t_{\sqrt{X}}$ (ns)	t_X (ns)
Q_0	5.16	35.56	35.56	35.56	35.56
Q_1	5.03	35.56	35.56	35.56	35.56
Q_2	4.86	35.56	35.56	35.56	35.56
Q_3	5.13	35.56	35.56	35.56	35.56
Q_4	5.16	35.56	35.56	35.56	35.56
Q_5	4.98	35.56	35.56	35.56	35.56
Q_6	5.16	35.56	35.56	35.56	35.56

TABLE IV. List of the single-qubit properties of ibm_perth which are open to the public [1]. The above data was taken at 07:32, 09/21/2023.

Qubit	ω_{01} (GHz)	t_{id} (ns)	t_{Rz} (ns)	$t_{\sqrt{X}}$ (ns)	t_X (ns)
Q_0	5.16	35.56	35.56	35.56	35.56
Q_1	5.03	35.56	35.56	35.56	35.56
Q_2	4.86	35.56	35.56	35.56	35.56
Q_3	5.13	35.56	35.56	35.56	35.56
Q_4	5.16	35.56	35.56	35.56	35.56
Q_5	4.98	35.56	35.56	35.56	35.56
Q_6	5.16	35.56	35.56	35.56	35.56

TABLE V. List of the single-qubit properties of ibm_perth which are open to the public [1]. The above data was taken at 04:30, 09/26/2023.

Qubits	t_{CX} (ns)
$[Q_0, Q_1]$ ($[Q_1, Q_0]$)	391.11 (426.67)
$[Q_1, Q_2]$ ($[Q_2, Q_1]$)	640.00 (604.44)
$[Q_1, Q_3]$ ($[Q_3, Q_1]$)	369.78 (334.22)
$[Q_3, Q_5]$ ($[Q_5, Q_3]$)	284.44 (320.00)
$[Q_4, Q_5]$ ($[Q_5, Q_4]$)	590.22 (625.78)
$[Q_5, Q_6]$ ($[Q_6, Q_5]$)	640.00 (604.44)

TABLE VI. List of the two-qubit properties of ibm_perth which are open to the public [1]. The above data was taken at 07:32, 09/21/2023.

Qubits	t_{CX} (ns)
$[Q_0, Q_1]$ ($[Q_1, Q_0]$)	391.11 (426.67)
$[Q_1, Q_2]$ ($[Q_2, Q_1]$)	640.00 (604.44)
$[Q_1, Q_3]$ ($[Q_3, Q_1]$)	369.78 (334.22)
$[Q_3, Q_5]$ ($[Q_5, Q_3]$)	284.44 (320.00)
$[Q_4, Q_5]$ ($[Q_5, Q_4]$)	590.22 (625.78)
$[Q_5, Q_6]$ ($[Q_6, Q_5]$)	640.00 (604.44)

TABLE VII. List of the two-qubit properties of ibm_perth which are open to the public [1]. The above data was taken at 04:30, 09/26/2023.
